# Kinetics of humoral immune response in patients with asymptomatic or mild COVID-19: a longitudinal study based in an in-house indirect ELISA method

**DOI:** 10.17179/excli2022-5337

**Published:** 2022-08-26

**Authors:** Nathanielly de Lima Silva, Danilo Nobre, Joyceane Alves de Oliveira, Márcia Santos Rezende, Joyce Thayane da Conceição dos Santos, Adriano Antunes de Souza Araújo, Lucindo José Quintans-Júnior, Rafael Ciro Marques Cavalcante, Luiz Carlos de Souza Ferreira, Paulo Ricardo Martins-Filho, Dulce Marta Schimieguel

**Affiliations:** 1 Department of Pharmacy, Laboratory of Hematology, Federal University of Sergipe, São Cristóvão, Sergipe, Brazil; 2 Graduate Program in Pharmaceutical Sciences, Federal University of Sergipe, Aracaju, Sergipe, Brazil; 3 Graduate Program in Health Sciences, Federal University of Sergipe, Aracaju, Sergipe, Brazil; 4 Laboratory of Neuroscience and Pharmacological Assays (LANEF), Federal University of Sergipe, São Cristóvão, Sergipe, Brazil; 5 Department of Pharmacy, Federal University of Sergipe, Lagarto, Sergipe, Brazil; 6 Institute of Biomedical Sciences, University of São Paulo, São Paulo, Brazil; 7 Investigative Pathology Laboratory, Federal University of Sergipe, Aracaju, Sergipe, Brazil

## ⁯

Studies have shown that severe acute respiratory syndrome coronavirus 2 (SARS-CoV-2) may induce humoral and cellular immune memory to receptor-binding domain (RBD), spike glycoprotein (S), and nucleocapsid (N) protein for at least five months in more than 90 % of individuals with COVID-19 (Dan et al., 2021[1]). However, there is consistent evidence for the effect of disease severity on antibody magnitude (Peluso et al., 2021[6]). It has been suggested that asymptomatic or mild COVID-19 patients have inadequate acquisition of humoral immunity (Takeshita et al., 2021[9]), which is critical for herd immunity and the development effective vaccination strategies. In this study, we evaluate changes in humoral immune responses in patients with asymptomatic or mild COVID-19 during six months of follow-up. 

In this longitudinal study, we selected 62 individuals (median age of 42.5 years; interquartile range [IQR] 33.3-52.0; 59.7 % female) tested positive for SARS-CoV-2 immunoglobulin G (IgG) antibodies using a fluorescence immunoassay (FIA) (iChroma II, BioSys) from July 2020 (peak of the first wave) to March 2021 (begging of the second wave associated with transmission of the Gamma variant) in the state of Sergipe, Northeast Brazil. Sergipe is located in a region with the worst socioeconomic indicators in the country, has an estimated population of 2.3 million, and a Human Development Index of 0.665. The first case of COVID-19 in Sergipe was confirmed on March 14, 2020, and at the time of writing this manuscript, more than 341,000 cases and 6400 deaths had been registered.

All participants included in the present study had a history of asymptomatic or mild COVID-19, were not vaccinated against the disease, and were selected from the EpiSERGIPE Project conducted in the state of Sergipe. The EpiSERGIPE project was a population-based serosurvey study that aimed to estimate the proportion of the population previously infected and to determine the population's level of immunity against SARS-CoV-2. In the state of Sergipe, the seroprevalence of SARS-CoV-2 antibodies in July 2020 and March 2021 was 9.3 % (95 % CI 8.5-10.1) (de Souza Araújo et al., 2021[2]) and 15.4 % (95 % CI 14.5-16.4) (unpublished data), respectively.

An in-house indirect enzyme-linked immunosorbent assay (ELISA)-based method was used to evaluate the serum titers of anti-nucleocapsid SARS-CoV-2 antibodies at three moments: baseline (D0), 90 days (D90), and 180 days (D180). Briefly, 96-microwell plates (Nunc, Thermo Fisher Scientific) were coated with 200 μl of recombinant SARS-CoV-2 nucleocapsid protein at 0.1 μg/mL and incubated overnight at 4 °C. On the following day, the plates were washed five times with 1 % PBS. Plates were blocked with 2 % BSA dissolved in PBS and incubated for 2 hours at 37 °C. The samples were diluted to 1:500 and incubated at 37 °C for 1.5 hours. Plates were washed five times with PBS-Tween (0.05 %) and goat anti-human IgG (HRP) secondary antibodies (Invitrogen, Thermo Fisher Scientific) were added at 1:20,000 and incubated at 37 °C for 1.5 hours. As previously, plates were washed five times and incubated for 20 min with 50 μl of o-phenylenediamine dihydrochloride (OPD) (SigmaFast OPD, Sigma-Aldrich). The reaction was stopped with 25 μl of 1M H_2_SO_4_ and read at 450 nm on a plate reader (Multiskan FC, Thermo Fisher Scientific).

To define the cut-off point, samples cryopreserved since the beginning of 2019 in our biobank, tested by fluorescent immunoassay and demonstrating a non-reactive result were later used as negative controls with a mean concentration of 3.59 ng/mL. Using the formula “cutoff=m+(2*SD)”, we obtained the final value of 11.49 ng/mL, where "m" represented the mean and "SD" the standard deviation of the concentrations of negative controls for SARS-CoV-2.

The serum titers of anti-nucleocapsid SARS-CoV-2 antibodies were plotted in boxplots. The assumptions of normality were checked, and the Friedman test and post hoc Conover's test (Bonferroni's correction) were used to compare the levels of IgG antibodies to the SARS-CoV-2 nucleocapsid protein between the three assessment periods. A two-way ANOVA was used to analyze the influence of sex (male and female) and age (≤ 40 years and > 40 years) on the levels of anti-SARS-CoV-2 antibodies. The significance level adopted was 5 %. The analyses were performed using the JASP software version 0.13 (JASP Team, Amsterdam, Netherlands).

In D0, 79 % (49 of 62) of individuals had a positive result for the presence of anti-nucleocapsid SARS-CoV-2 antibodies. In D90 and D180, the percentage of positive results was 69.3 % (43 of 62) and 53.2 % (33 of 62), respectively. We found a progressive decline in the levels of anti-nucleocapsid SARS-CoV-2 antibodies over time, ranging from 26.2 (IQR 12.4-37.7) in D0 to 11.7 (IQR 5.6-18.2) in D180 (p < 0.001) (Figure 1; supplementary file). In addition, we found that individuals over 40 years old had higher levels of IgG antibodies to the SARS-CoV-2 nucleocapsid in D90, regardless of sex. An influence of sex and age on the humoral response was not observed on D0 and D180 (Figure 2; supplementary file).

Asymptomatic or mild disease has been found in about 80 % of patients with SARS-CoV-2 infection. In most cases, antibody levels increase within the first weeks of infection (Qu et al., 2020[7]), but our results showed that about 20 % of individuals did not seroconvert in the first evaluation. Although clinical and biological characteristics may influence the individual immune response, it is possible that this negative seroconversion rate is a result of inherent limitations of the immunoassay, including its sensitivity and the use of only one target SARS-CoV-2 protein. Furthermore, despite studies having suggested that antibody titers are determined especially by disease severity and not duration (Seow et al., 2020[8]), we found a progressive decline in the levels of anti-nucleocapsid SARS-CoV-2 antibodies and an approximately 30 % decrease in the number of individuals with detectable IgG antibodies six months after an asymptomatic or mild SARS-CoV-2 infection. 

The present findings suggest that the natural humoral response after SARS-CoV-2 infection in outpatients is not sustained in the long term, which may increase the risk of reinfection for these individuals. In a study performed by Lumley et al. (2021[3]), it was shown that previous SARS-CoV-2 infection is associated with protection from reinfection for most people for at least six months. The decline in humoral response after asymptomatic or mild infection has been associated with lower viral load and lower release of pro-inflammatory cytokines, as well as a decrease in blood B-cell ratio and antibody-secreting cells (Yang et al., 2021[10]). 

Age and sex are factors that can influence the humoral response through antibody production during and after SARS-CoV-2 infection. In our study, we found that subjects over 40 years old had higher levels of IgG antibodies to the SARS-CoV-2 nucleocapsid than those younger subjects three months after infection. In addition, we found no differences between sexes regarding the serum titers of anti-nucleocapsid SARS-CoV-2 antibodies at the three time points. These findings showed different kinetics in the humoral response against SARS-CoV-2 infection according to age and corroborate other studies that showed that older age is associated with sustained antibody response in COVID-19 (Noh et al., 2021[5]). Although the impact of age on COVID-19 clinical outcomes is recognized, it has been suggested that immunosenescence could be two forces with opposite effects, partly explaining the complex relationship between age and antibody levels after COVID-19 infection (Meyer et al., 2022[4]).

This study showed a progressive decline in the levels of anti-nucleocapsid SARS-CoV-2 antibodies during the first six months after SARS-CoV-2 infection among individuals with asymptomatic or mild disease. Approximately 50 % of individuals have no detectable antibodies six months after infection. Moreover, we found a potential influence of age on the humoral response against SARS-CoV-2. 

## Declaration

### Authors' contributions

Silva NL, Nobre D, de Oliveira JA, Rezende MS, dos Santos JTC: collected and analyzed the data; interpreted the results; writing − review and editing; De Souza Araújo AA, Quintans-Júnior LJ, Martins-Filho PR, Cavalcante RCM, Ferreira LCS, Schimieguel DM: conceptualisation; project administration; resources; interpreted the results; drafted the manuscript; writing - review and editing.

### Conflict of interest

The authors have no conflicts of interest to declare.

### Role of funding source

This study is part of the EpiSERGIPE project which is supported by grant SES/FAPESE/UFS 001/2020.

### Acknowledgments

We thank the healthcare workers and members of the EpiSERGIPE project for their efforts in the fight against COVID-19 in the state of Sergipe.

## Supplementary Material

Supplementary file
