# Supplementary material for: Kinetics of humoral immune response in patients with asymptomatic or mild COVID-19: a longitudinal study based in an in-house indirect ELISA method
Source: EXCLI J. 2022 Aug 26;21:1167–70. doi: 10.17179/excli2022-5337 (PMC9650694; doi:10.17179/excli2022-5337)
Supplement: Supplementary file [file EXCLI-21-1167-s-001.pdf]

EXCLI Journal 2022;21:1167-1170 – ISSN 1611-2156
Received: August 16, 2022, accepted: August 23, 2022, published: August 26, 2022

Supplementary file to:

Letter to the editor:

KINETICS OF HUMORAL IMMUNE RESPONSE IN PATIENTS WITH ASYMPTOMATIC OR MILD COVID-19: A LONGITUDINAL STUDY BASED IN AN IN-HOUSE INDIRECT ELISA METHOD

Nathanielly de Lima Silva[1,2], Danilo Nobre[1], Joyceane Alves de Oliveira[1,2], Márcia Santos Rezende[1,2], Joyce Thayane da Conceição dos Santos[1,2], Adriano Antunes de Souza Araújo[1,2,3], Lucindo José Quintans-Júnior[2,3,4], Rafael Ciro Marques Cavalcante[5], Luiz Carlos de Souza Ferreira[6], Paulo Ricardo Martins-Filho[7], Dulce Marta Schimieguel[1,2]

1 Department of Pharmacy, Laboratory of Hematology, Federal University of Sergipe, São Cristóvão, Sergipe, Brazil
2 Graduate Program in Pharmaceutical Sciences, Federal University of Sergipe, Aracaju, Sergipe, Brazil
3 Graduate Program in Health Sciences, Federal University of Sergipe, Aracaju, Sergipe, Brazil
4 Laboratory of Neuroscience and Pharmacological Assays (LANEF), Federal University of Sergipe, São Cristóvão, Sergipe, Brazil
5 Department of Pharmacy, Federal University of Sergipe, Lagarto, Sergipe, Brazil
6 Institute of Biomedical Sciences, University of São Paulo, São Paulo, Brazil
7 Investigative Pathology Laboratory, Federal University of Sergipe, Aracaju, Sergipe, Brazil

* **Corresponding author:** Dra. Dulce Marta Schimieguel, Departamento de Farmácia, Laboratório de Hematologia, Universidade Federal de Sergipe, São Cristóvão, Sergipe, Brasil. CEP: 49100-000. E-mail: dulcemarta@hotmail.com

https://dx.doi.org/10.17179/excli2022-5337

This is an Open Access article distributed under the terms of the Creative Commons Attribution License (http://creativecommons.org/licenses/by/4.0/).

EXCLI Journal 2022;21:1167-1170 – ISSN 1611-2156
Received: August 16, 2022, accepted: August 23, 2022, published: August 26, 2022

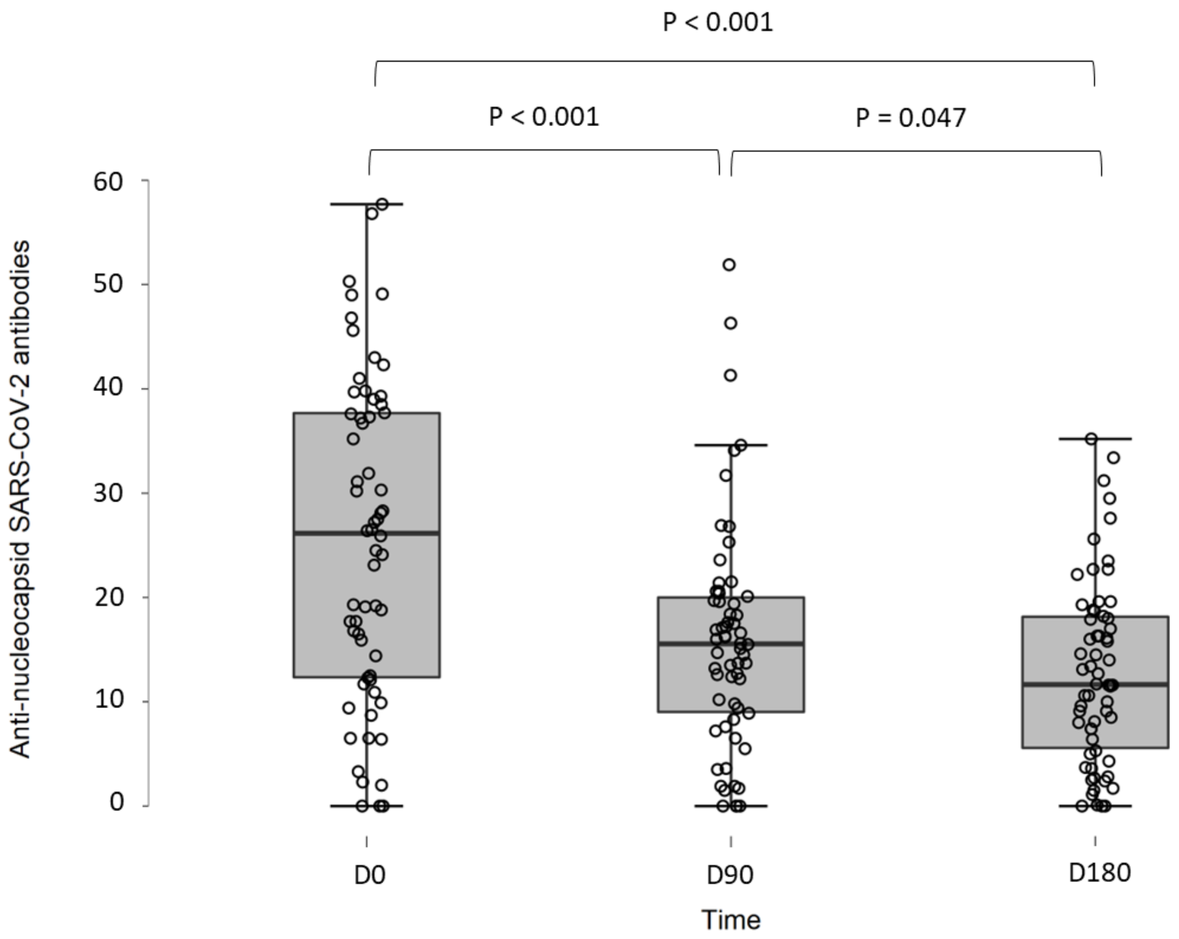

Figure 1: Natural humoral response in patients with asymptomatic or mild COVID-19

EXCLI Journal 2022;21:1167-1170 – ISSN 1611-2156
Received: August 16, 2022, accepted: August 23, 2022, published: August 26, 2022

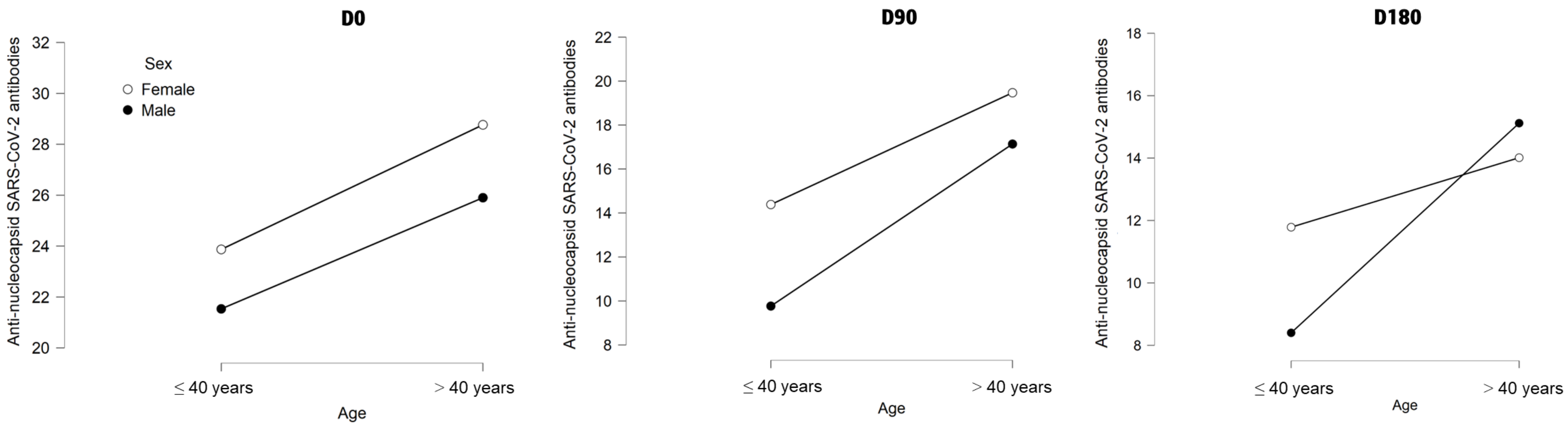

D0

Variables	P-value
Sex	0.530
Age	0.265
Sex * Age	0.949

D90

Variables	P-value
Sex	0.222
Age	0.031
Sex * Age	0.686

D180

Variables	P-value
Sex	0.625
Age	0.059
Sex * Age	0.337

Figure 2: Influence of age and sex on the humoral response in patients with asymptomatic or mild COVID-19
